# Parents’ stress and distress in the face of their children’s self-injurious thoughts and behaviors: a scoping review

**DOI:** 10.1007/s00787-025-02886-4

**Published:** 2025-11-06

**Authors:** Or Cohen Ben Simon, Shimrit Daches, Yari Gvion

**Affiliations:** Department of Psychology, Bar-Ilan University, Ramat Gan, Israel

**Keywords:** Parent, Stress, Distress, Youth, Self-harm, Suicide

## Abstract

Children’s and youths’ self-injurious thoughts and behaviors (SITBs) significantly impact their parents, often causing elevated stress and distress. In addition to managing the emotional toll, parents need to actively participate in their child’s recovery, adding a second layer of caregiving burden. Despite the clinical relevance of this issue, few quantitative studies have examined parental stress during their child’s suicidal crisis, and the nature of this association remains insufficiently understood. To address this, we conducted a scoping review to identify, summarize, and synthesize quantitative studies linking children’s and youths’ SITBs with parental stress and/ or distress. We systematically searched three databases for research published on or before May 2023. Studies were eligible if they provided data on the association or impact of children’s and youths’ SITBs on parental stress and/ or distress levels, including intervention studies that reported outcomes related to parental distress. The findings of this review highlight three key insights: (1) Descriptive data suggest that parental stress and/ or distress is significant among parents of children and youths exhibiting SITBs, even when not reaching the clinical threshold for psychopathology; (2) Parents of children and youths with SITBs report significantly higher levels of stress and distress compared to parents in control groups; (3) Parents benefit from various therapeutic interventions. This scoping review underscores the importance of investigating the nature and magnitude of the association between children’s and youths’ SITBs and parental stress and distress, as well as the outcomes of targeted interventions.

## Introduction

Suicide is the fourth leading cause of death among youth [1]. Data from recent years have shown an increase in the number of youths arriving at emergency rooms (ERs) with suicide-related concerns [2–4]. Suicidal ideation (SI), non-suicidal self-injury (NSSI), deliberate self-harm (DSH), and suicide attempts (SAs) are all forms of self-injurious thoughts and behaviors (SITBs). While these behaviors are grouped under the umbrella of SITBs, it is important to note that they differ in intent: NSSI refers to the deliberate infliction of harm on oneself without the intention to die, whereas suicidal behaviors involve thoughts of death, planning, or actions taken with the intent to die [5]– [6]. This review includes studies examining both suicidal and non-suicidal forms of self-injury, given their shared emotional and behavioral contexts and their potential impact on parents. When SITBs occur in children and youth, they not only pose a significant risk to the young individuals themselves but also have a profound impact on their parents, often leading to heightened stress and distress [7–9]. Qualitative studies have indicated that parents of children and/or youth who experience SITBs feel overwhelmed and undergo recurring emotional turmoil [10]– [11]. This turmoil encompasses a spectrum of negative emotions, including guilt, self-blame, shame, fear, shock, anger, and helplessness [8, 10, 12]. Additionally, parents often experience anxiety regarding the unforeseen long-term consequences of their child’s mental health, which intensifies, particularly when their child experiences SITBs [8, 12–14].

Building on theoretical frameworks of parent-child reciprocity [15] and transactional models [16], research has explored the influence of children’s and youths’ psychopathology on parental mental health. Studies have shown that when children and youth experience mental health challenges, their parents are also at heightened risk for psychological distress. For instance, longitudinal findings from large-scale national surveys indicate that parents whose child develops emotional disorders are more likely to experience deteriorating mental health over time [17]. Similarly, Raposa et al. [18] found that youth psychopathology conferred a risk for parents’ current and future depression, independent of their prior mental health status. These findings highlight the importance of viewing parents not merely as caregivers responsible for their child’s treatment but also as individuals whose well-being is deeply affected by their child’s psychological challenges. While these studies have addressed parents of youth with various mental health conditions, SITBs represent a unique clinical crisis due to their direct threat to the youth’s life.

Examining parallels between parents of youth experiencing SITB and those of children and youth with life-threatening physical illnesses further underscores the psychological toll on caregivers. When children and youth are admitted to a pediatric intensive care unit or ER due to life-threatening physical illnesses, parental stress levels escalate, and, in certain instances, psychopathological symptoms may emerge [19]. Across studies, approximately 23–45% of parents of critically ill children report elevated anxiety symptoms [20], 13–74% grapple with depression [20]– [21], 10–37% face post-traumatic stress disorder (PTSD) [20, 22–24], and 30–84% experience subclinical PTSD [22]– [23]. Other research on parents of critically ill children and youth has shown that they contend with sleep problems and issues in family functioning [25–29]. Parental symptoms may persist for months after their child’s hospital discharge [30]– [31].

In contrast to parents of children and youth with mental illness, which often does not pose a threat to their lives, or parents of children and youth with life-threatening physical illnesses, parents of children and youth experiencing SITBs are required to play a highly active role in their child’s recovery process and suicide prevention [32–35]. When their child engages in SITBs, parents are expected to prevent and minimize suicidal risk by implementing various crisis interventions (e.g., suicidal ideation monitoring; [35]) and to bear the responsibility of restricting access to lethal means to prevent their child’s suicide [34]. These caregiving expectations, coupled with the life-threatening nature of suicidality, may heighten parental stress and distress, leading to psychopathological symptoms [8, 14, 35].

Whereas parents’ adjustment to their child’s life-threatening (physical) illnesses has been widely discussed and conceptualized (see the Integrative Model of Pediatric Medical Traumatic Stress [36]), the adjustment process of parents facing their child’s suicide risk has received limited quantitative attention. Although several studies have reviewed the literature on this topic [11, 37], to the best of our knowledge, a comprehensive overview and synthesis of the quantitative literature appears to be missing. There is a clear need to quantify the unique association between children and youth’s SITBs and parental stress and distress levels in order to establish suitable sources of help and support. Given the limited number of studies employing quantitative methodologies, we conducted a scoping review to examine the association between children’s and adolescent’s SITB and parental stress and/or distress levels. Our goal was to summarize and integrate existing literature on this association, with the aim of informing clinical practice and guiding future research in this field.

## Method

As the evidence base regarding the link between children and youth’s SITBs and their parents’ stress and/or distress levels is lacking, a scoping review was deemed appropriate to map the current literature. We conducted this review to examine the extent, range, and nature of research activity and to identify gaps in the existing literature. The current study was guided by the review methods outlined by Levac et al. [38], which are based on the Arksey and O’Malley [39] framework.

### Search strategy

A broad electronic search strategy was employed to identify peer-reviewed studies exploring the association between children and/or youth’s SITBs and/or depression and parental stress and/or distress levels. The search adhered to established PRISMA principles [40]– [41]. PubMed, PsycNET, and Google Scholar databases were searched. Search terms entered into PubMed and PsycNET were (“parent” OR “caregiver stress/distress” OR “mother stress/distress” OR “father stress/distress " OR “maternal stress/distress " OR “paternal stress/distress”) AND (“child” OR “teen” OR “youth” OR “adolescent” OR “pediatric” OR “juvenile” OR “children”) AND (“suicide” OR “suicide ideation” OR “suicidal ideation” OR “suicidal behavior” OR “non-suicidal self-injury” OR “deliberate self-harm” Or “depression” OR “affective disorder”). In addition, a Google Scholar search included the terms “parent* stress” OR “parent* distress” AND “suicide” AND “adolescent.”

### Inclusion/exclusion criteria

Studies were considered eligible for inclusion if they were written in English, peer-reviewed, and provided data on the association between, and/or effects of, children’s and youth’s (aged up to 24 years) SITBs on parental stress and/or distress levels. Our inclusion criteria encompassed studies in which SITBs were the primary focus or clearly examined as a unique variable. Similarly, intervention studies involving children and youth experiencing SITBs were included if they reported such data as well. The search for studies concluded in May 2023. Case studies, reviews, books, book chapters, conference papers, non-English papers, qualitative studies, incomplete studies, and clearly irrelevant studies were excluded. Additionally, studies in which parental stress or distress were examined as predictors of children’s or youths’ SITBs were excluded, unless they provided specific descriptive data on parental stress or distress or reported correlational data between child or youth suicide risk and parental stress or distress levels.

In cases where the relevance of a particular study was unclear from the abstract, the full text was reviewed for further clarification. All studies were meticulously assessed against the inclusion and exclusion criteria by the first author and three research assistants. Any disputed studies were discussed until a consensus was reached. A total of 25 studies met all the inclusion criteria listed in Table 1.

**Table 1:** Study characteristics and relevant main findings

	Author and year		Pub. year	Methodology	Country	Parents (*N*)		Offspring ages	Child illness (type)	Clinical measure evaluating child’s suicide risk (reported by child/parent/clinician)	Clinical measure evaluating parental stress and distress	Descriptive information	Main results
1	James et al.	2023		Cross-sectional, Physiology	USA		*N* = 60	13–17	NSSI	*Child*: SITBI	Facial electromyography data, BDI- II, BAI	For mothers of adolescents with a history of NSSI, the mean level of BDI-II^b^ score was M = 10.38 (SD = 8.79), and the mean level of BAI^c^ score was M = 10.38 (SD = 8.79).	Mothers of adolescents with a history of NSSI exhibited elevated levels of negative facial affect, felt increased anxiety following conflict task, and were slower to return to their positive facial affect setpoint.
2	Sheftall et al.	2021		Cross-sectional	USA		*N* = 117	6–9	SI	*Parent and child*: C-SSRS	BSI	BSI^e^ global severity index mean score among parent to children with SI was M = 1.3 (SD = 0.7)	Parent BSI^e^ score did not differ between groups (MH vs. SI+).
3	Weinstein et al.	2015		Cross-sectional	USA		*N* = 72 parent or caregiver	7–13	Bipolar I, II, or not otherwise specified (NOS), SI	*Child*: C-SSRS^d^	PSS	PSS-18^f^ mean scores ranged between M = 37.29–53.50 (SD = 8.22–18.73).	Parenting PSS-18^f^ score varied by group: parents of youth with active ideation with method/some intent reported greater stress than parents of youth with nonspecific active ideation.
4	Greening et al.	2010		Cross-sectional	USA		*N* = 172	6–12	Suicidal behavior, depressive symptoms, and proactive and reactive aggression	*Child*: RSQ	HSCL	Caregivers’ mean score on the HSCL-58^h^ was M = 44.86 (SD = 29.95).	Children with higher total RSQ^g^ score also had parents who reported more parental distress. However, children’s specific score (sub-scale) for suicide ideation/behavior did not correlate with parental distress.
5	*Barksdale et al.*	2009		Cross-sectional	USA		*N* = 1854 parent (78.6%) or caregiver	5–22	SA	*Parent*: responded to two structured interview questions concerning their child’s history of suicide attempts.	CGSQ		Lifetime SA was related to caregiver strain. Differences in subjective internalizing and objective strain were evident between caregivers of youth with no attempts versus single and multiple attempts.
6	Whitlock et al.	2017		Mixed method	USA		*N* = 253	13–24	NSSI	*Parent*: Items assessing NSSI-specific characteristics included whether the child was still engaged in NSSI behavior (yes, no, or I do not know) and the length of time injuring.	CGSQ^i^		The difference between parent groups (NSSI + vs. MH) remained significant for all types of caregiver strain.
7	Townsend et al.	2021		Mixed method	Australia		*N* = 37 parent or foster carer	12–18	DSH	*Parent*: Parents were asked to provide information regarding their adolescent who self-harmed.	MHI-5	MHI-5^j^ mean score was M = 50.96 (SD = 20.50). 66.7% of the sample were categorized as likely to have mental illness.	Parents reported reduced capacity to function and difficulties conducting their work or activities.
8	Rotheram-Borus et al.	1996		Intervention	USA		*N* = 140	12–18	SA	*Clinician*: PSIS *Child*: BDI, HASS	BDI^l^, BSI^e^	Mothers’ mean BDI^l^ levels ranged between M = 9.7–13.1 (SD = 7.90–10.7), and mean BSI^e^ (global severity index) levels ranged between M = 26.5–38.8 (SE = 26.6–34.3).	Mothers in the specialized program reported lower levels of depression on the BDI^l^ and less overall psychopathology on the BSI^e^.
9	Rotheram-Borus et al.	2000		Intervention	USA		*N* = 140	12–18	SA	*Child*: The Suicidal Ideation Scale taken from HASS^m^	BDI^l^, SCL- 90, BSI^e^	Mothers’ mean BDI^l^ score was M = 11.5,(SD = 9.4). Mothers’ mean BSI score was M = 0.63 (SD = 0.6).	At the post-ER assessment, mothers in the specialized ER care condition reported lower scores on the BDI^l^ and this difference persisted over 18 months.
10	Woodberry and Popenoe	2008		Intervention	USA		*N* = 19 parent or caregiver	13–18	SA, DSH and/intense and unstable affect or relationships	*Child*: RADS	BDI^l^	Parents’ mean BDI^l^ at baseline was M = 10.68 (SD = 11.06). At all time- points, parents depressive symptoms were below the clinical cutoff.	Statistically significant improvements were observed in parent depressive symptoms from pre to post treatment.
11	Asarnow et al.	2011		Intervention	USA		*N* = 181	10–18	SI and/SA	*Child/Parent*: DISC-IV, HASS^m^, CES-D	CES-D^q^	CES-D^q^ mean scores at baseline M = 19.44 (SD = 13.46).	Statistically significant improvements were observed in parent depressive symptoms (CES-D^q^) from baseline to follow-up.
12	Asarnow et al.	2015		Intervention	USA		*N* = 35	11–18	SA	*Clinician*: NIMH DISC IV^p^ *Child*: C-SSRS^d^, HASS^m^	CES-D^q^	CES-D^q^ mean scores from baseline to follow-up were M = 20.26–10.86 (SE = 13.34–8.55).	Statistically significant improvements were observed in parent depressive symptoms from baseline to follow-up.
13	Babeva et al.	2020		Intervention	USA		*N* = 50	11–18	NSSI, SA	*Clinican*: NIMH DISC-IV^p^ *Child*: HASS^m^, CES-D^q^	CES-D^q^	Clinically elevated depressive symptoms were reported in 62% of parents at baseline.	Results indicated large effect sizes for SAFETY on parental depression.
14	Wijana et al.	2018		Intervention	Sweden		*N* = 49	13–19	DSH	*Child*: DSHI	PSS-10 HADS	Mothers’ baseline mean depression score was M = 8.22, (SE = 0.31) mean anxiety score was M = 11.73, (SE = 0.30), mean stress (PSS-10^s^) score was M = 23.98, (SE = 0.86). Fathers’ baseline mean depression score was M = 7.43 (SE = 0.44) anxiety score was M = 9.84 (SE = 0.24), stress score was M = 19.55 (SE = 1.05).	Mothers and fathers reported reduced levels of stress from pre- to post-treatment. Mothers experienced reduced levels of anxiety across treatment, while fathers’ levels of anxiety increased from pre- to post-treatment.
15	Cloutier et al.	2021		Intervention	Canada		*N* = 30 parent or caregiver	13–17	SI	*Child*: SIQ-JR	PSS-10^s^	PSS-10^s^ mean baseline score was M = 21.3 (SD 7.0).	Parents had reduced perceived stress across treatment.
16	Gillespie et al.	2019		Intervention	Ireland		*N* = 167 parent or legal guardians	13–18	DSH or suicidal act having occurred in the past 16/24 weeks or chronic SI reported	*Child*: Questionnaire for suicidal ideation. A 6-item measure developed by the research team.	BAS, PSS^f^	Parents’ mean BAS^u^ baseline scores ranged between M = 45.55–49.49 (SE = 11.95–9.78). PSS-18^f^ mean baseline scores ranged between M = 41.92–42.51 (SE = 11.49–10.01).	Significant reductions were reported from pre- to post-intervention on all outcome measures for adolescents and parents, for the 16- and 24-week program, respectively.
17	Power et al.	2009		Intervention	Ireland		*N* = 46	16 years and under	Engaged in or expressed thoughts of DSH	*Parent*: SDQ	GHQ-12	GHQ-12^x^ mean score at time 1 was M = 6.33. A high number of parents met the criteria for psychological distress (76%) at Time 1.	Parents’ psychological distress was reduced after the program.
18	Cotrell et al.	2018		Intervention	UK		*N* = 832 parent or caregiver	11–17	DSH	*Child: SASII*, *BSS* *Clinician: CDRS*	GHQ-12^x^	GHQ-12^x^ mean score was M = 18.2 (SD = 7.16)	Psychological distress was significant. There was no significant difference at parent GHQ-12^x^ scores between the two types of treatment 12 and 18 month after the intervention.
19	Asarnow et al.	2021		Intervention	USA		*N* = 173	12–18	SA, DSH, and SI	*Child*: SASII^y^	BSI^e^, BSL-23	Parent BSI^e^ (global severity index) mean baseline scores were M = 0.59–0.62 (SE = 0.06), and parent BSL-23 mean score at baseline was M = 0.66 (SE = 0.06).	Parent BSL-23^bb^ and BSI^e^ improved after treatment and across the 12-month period.
20	Perloe et al.	2014		Intervention	USA		*N* = 241	12–18	Depressive symptoms and SI	*Child*: CDRS^aa^, SIQ-JR^u^	BDI-II^b^	Mothers’ mean BDI-II^b^ at baseline was M = 10.43, (SE = 9.7). At all time- points, maternal depressive symptoms were below the clinical cutoff.	Trajectories of maternal depression and adolescent suicidal ideation did not fluctuate together. Initial levels of maternal depressive symptoms and adolescent suicidal ideation were not correlated significantly. Maternal depressive symptoms decreased across treatment.
21	Asarnow et al.	2017		Intervention (**Parents were assessed once)	USA		*N* = 42	12–18	DSH/SA	*Child*: C-SSRS^d^, SHI, CES-D^q^	CES-D^q^	Clinically elevated depressive symptoms were reported in 52.4% of primary caretaking parents (mostly mothers, 85.7%) at baseline.	No pre-to-post results were provided for the parents.
22	Morgan et al.	2013		Intervention (**Parents were assessed once)	Ireland		*N* = 130	18 years and under	DSH and suicidal behavior	*Parent*: SDQ^w^	GHQ-12^x^	Most parents (86%) met the criteria for psychological distress.	There was a small positive correlation between parental well-being and reported child difficulties.
23	Smith et al.	2023		Longitudinal (2 weeks follow-up)	USA		*N* = 118 parent or legal guardians	10–17	SI and suicide behavior	*Child*: SIQ-JR^u^	PHQ-4, PANAS, ISI	PHQ-4^dd^ depression and anxiety baseline scores and 2 weeks later scores: M = 3.19 (SE = 3.09); M = 5.4 (SE = 3.6). The ISI baseline score and 2 weeks later scores: M = 7.74 (SE = 6.21); M = 10.05, (SE = 5.8), PANAS^ee^ negative subscale baseline score and 2 weeks later scores: M = 21.38 (SE=. 8.14); M = 23.19 (SE = 8.96).	Parents PHQ-4^dd^ total scores and ISI^ff^ scores increased between youths’ ED visit and 2-week follow-up. There was no significant change over time in caregiver negative affect. Parent mental health history and youth suicide chronicity impacted distress.
24	*Kandsperger et al.*	2022		Longitudinal (**Parents stress levels were assessed once)	Germany		*N* = 96 parent or caregiver	11–19	Self-injurious thoughts and behaviors	*Child*: SITBI^a^	EBI		Youth’s NSSI was not associated with parent’s stress levels.
25	Ewell Foster et al.	2021		Longitudinal (**Parents stress and distress were assessed once)	USA		*N* = 118 parent or legal guardians	10–17	Depressive symptoms, NSSI, SI, SA	*Child*: SIQ-JR^u^	PHQ-4^dd^, ISI^ff^, Negative Affect subscale of the PANAS^ee^	PHQ-4^dd^ total mean score was M = 3.38 (SD = 3.4), ISI^ff^ mean score was M = 7.89, (SD = 6.6), and PANAS^ee^ mean negative affect was M = 20.74 (SD = 8.18).	The frequency of stressors was significantly correlated with increased insomnia severity and negative affect.

*SITBI* Self-Injurious Thoughts and Behaviors Interview, *BDI-II* Beck Depression Inventory-II, *BAI* Beck Anxiety Inventory, *C-SSRS* Columbia Suicide Severity Scale, *BSI* Brief Symptom Inventory, *PSS* Parenting Stress Scale, *RSQ* Risk for Suicide Questionnaire, *HSCL* Hopkins Symptom Checklist, *CGSQ *Caregiver Strain Questionnaire, *MHI-5* Mental Health Inventory 5, *PSIS* Patient Safety Indicators, *BDI *Beck Depression Inventory, *HASS *Harkavy Asnis Suicide Survey, *SCL* Symptom Check List, *RADS* The Reynolds' Adolescent Depression Scale, *DISC-IV* Diagnostic Interview Schedule for Children and Adolescents, *CES-D* Center for Epidemiological Studies Depression Scale, *DSHI* Deliberate Self-Harm Inventory, *PSS-10* Perceived Stress Scale, *HADS* Hospital Anxiety and Depression Survey, *SIQ-JR* Suicidal Ideation Questionnaire-Junior Revised, *BAS* Burden Assessment Scale, *SDQ* Strength and Difficulties Questionnaire, *GHQ-12 *General Health Questionnaire, *SASII *Suicide Attempt Self-Injury Interview, *BSS *Beck Scale for Suicide Ideation, *CDRS* Clinician Depression Rating Scale-Revised, *BSL *Borderline Symptom List, *SHI *Suicide History Interview, *PHQ-4* Four-item Patient Health Questionnaire, *PANAS* Positive and Negative Affect Schedule, *ISI *Insomnia Severity Index, *EBI *The German Version of the Parental Stress Index

### Methodological factors

## Results

### Overview of included studies

Twenty-five studies were identified as reporting findings on parental stress and/or distress levels in the context of a child’s or youth’s SITBs (see Table 1). The studies originated from seven countries: USA (*n* = 17), Ireland (*n* = 3), Australia (*n* = 1), Canada (*n* = 1), Germany (*n* = 1), England (*n* = 1), and Sweden (*n* = 1). Most studies included parents of youth aged between 10 and 19 (*n* = 18); three studies focused on parents of younger children aged 6–13 (*n* = 3); and the rest (*n* = 4) included a broad age range (<18; <16; 5–22; 13–24). Most studies (*n* = 14, 56%) were published between 2017 and 2023; the rest were published between 1996 and 2016. Most studies (*n* = 15) included only parents; however, some studies (*n* = 10) also included a small number of caregivers who were not parents but served as caregivers for the child.

Fifteen studies (60%) examined the mental health or related outcomes of children and youth, considering parental stress as an independent variable, a secondary or exploratory outcome, or a covariate [7, 42–55]. Six studies (24%) focused on parental stress and/or distress; five examined parental stress and/or distress levels as the dependent variable [14, 56–60]. Additionally, four studies (16%) addressed both parents’ and child’s mental health to a similar extent [35, 61–63].

Parental stress and/or distress were measured using self-report measures in all studies, with one study incorporating both self-report and physiological measures [62]. The most used self-report measure was the Beck Depression Inventory/BDI [64], (*n* = 5, 20%). The second most frequently used self-report measures were the Center for Epidemiological Studies Depression Scale/CES-D [65] (*n* = 4, 16%) and the Brief Symptom Inventory/BSI [66], (*n* = 4, 16%). The General Health Questionnaire/GHQ-12 [67], (*n* = 3, 12%) was used in three studies. The Parenting Stress Scale/PSS [68], the Perceived Stress Scale/PSS [69], the four-item Patient Health Questionnaire/PHQ-4 [70], the Insomnia Severity Index/ISI [71], the negative affect subscale of the Positive and Negative Affect Schedule/PANAS [72], and the Caregiver Strain Questionnaire/CSQ [73] were all used twice. Other measures were used only once.

### Research questions and findings related to parental stress and/or distress in the face of their child’s SITBs

Descriptive information about parental stress and/or distress, as well as the main findings of each study, are presented in Table 1. Data from 22 studies provided descriptive accounts of parental stress and distress levels. Studies utilizing general health measures reported that 66.7% to 86% of parents met the criteria for psychological distress [57]– [58]. Two studies reported moderate stress levels among parents [44, 63]. Also, two studies reported above cutoff depressive symptoms at baseline [42, 52], while two other studies reported that 52% to 62% of parents experienced clinically elevated depressive symptoms, indexed by the CES-D [7, 54]. In contrast, five studies revealed minimal-mild depression levels among parents, indexed by the BDI [48, 51, 55, 61]– [62]. Wijana et al. [63] reported that depressive symptoms among mothers were suggestive of the presence of depression, while fathers showed non-significant levels of depression, as measured by the Hospital Anxiety and Depression Scale (HADS). Differences in anxiety levels were also observed, with mothers exhibiting probable (‘caseness’) levels of anxiety, whereas fathers reported anxiety levels that were only suggestive of its presence. James et al. [62] reported mild anxiety symptoms among mothers, indexed by the BAI [74]. Two additional studies reported general anxiety and depressive symptom scores using the PHQ-4, both indicating mild symptom levels [35, 59]. Ewell-Foster et al. [35] reported that 16.8% of parents experienced moderate to severe insomnia, while Smith et al. [59] reported that parents’ insomnia was sub-threshold.

The studies reviewed were grouped according to three primary purposes: (1) Exploring the strength and nature of the association between children’s and youths’ SITBs and parental stress and/or distress symptoms; (2) Assessing differences in stress and/or distress levels between parents of children and youths who engage in SITBs and parents in a comparison group; (3) Evaluating the effectiveness of support programs or therapeutic interventions (SAFETY; Supporting Parents and Carers of Young People with Self-Harm/SPACE; Dialectical Behavioral Therapy/DBT; Building Resilience and Attachment in Vulnerable Adolescents/BRAVA; Treatment of Resistant Depression in Adolescents/TORDIA; Family Intervention for Suicide Prevention/FISP; family therapy; specialized ER care) on parental stress and/or distress symptoms.

### Exploring the strength and nature of the association between child’s SITBs and parental stress and/or distress symptoms

Six studies reported at least one finding on the association between children’s and youths’ SITBs and parental stress and/or distress symptoms [46]– [47, 50, 58]– [59, 61]. Weinstein et al. [50] conducted a cross-sectional study among 72 children and youths diagnosed with bipolar I, II, or not otherwise specified (aged 7–13) and their parents/caregivers. They examined the association between a child’s SI and parenting stress. Study results indicated that parents of children and youths with specific active suicidal ideation or some suicidal intent reported significantly more stress than parents of children and youths with nonspecific active ideation. Similarly, Power et al. [58], conducted an intervention study involving 46 parents of children and youths (aged 16 and under) with a previous history of DSH, and reported a significant positive association between child’s history of repeated DSH and parental distress.

The results of Greening et al. [46] and Smith et al. [59] were less conclusive. The Greening et al. [46] cross-sectional study, which involved 173 children and youth aged 6–12 years and their parents, reported on the association between the child’s suicidal behavior and parents’ emotional and somatic distress. A correlation analysis revealed that the child’s general suicidal behavior severity score, according to the Risk for Suicide Questionnaire total score [75], was positively associated with parental distress. However, children and adolescents SI and SBs (sub-score computed separately) did not correlate with parental distress. The Smith et al. [59] longitudinal study, conducted more recently, described the course of caregiver distress symptoms (e.g., anxiety, depression, and negative affect) and sleep problems following their child’s suicide-related ER visit and two weeks later. This study involved 118 parents of youth aged 11–17 and found that parents experienced a significant increase in symptoms of anxiety, depression, and sleep problems two weeks after their child’s suicide-related ER visit (compared to at the time of the ER visit itself). Furthermore, they found that parents of youth with a prior ER visit for psychiatric concerns reported higher overall anxiety and depressive symptoms at follow-up. However, no significant changes were observed in parents’ negative affect over time, and the severity of the youth’s SI did not predict parent outcomes two weeks later.

The studies by Kandsperger et al. [47] and Perloe et al. [61] did not find a significant association between child and youth SITB and parental stress or distress. However, both studies reported associations between the child’s scores on other mental health measures and parental stress. Kandsperger et al. [47], for example, assessed 96 parents of youths aged 11–18 years who engaged in NSSI. The authors found that the youth’s NSSI (duration, lifetime prevalence) was not associated with parental stress levels. However, the youth’s score on the Clinical Global Impression Scale was significantly related to some aspects of parental stress (but not all). Furthermore, Perloe et al. [61] conducted an intervention study involving 241 mothers and youths (aged 12–18) that examined the association between youth depression/SI and maternal depression over time and across treatment. The results indicated that initial levels of youth depressive symptoms and SI were not significantly correlated with initial maternal depressive symptoms. However, Perloe et al. [61] found an association between youth depression (but not SI) and maternal depression over a 72-week study period and across treatment.

To summarize, the results regarding the association between children and youth SITBs and parental stress and distress are inconclusive. Two studies reported a significant and clear association between children and youth SITBs and parental stress and distress [50, 58]. Two other studies found a partial association or reported evidence that could imply such an association [46, 59]. The last two studies [47, 61] failed to find an association between children and youth SI and NSSI and parental stress and distress, though they did report an association between other mental health measures of the child and parental stress or distress. Due to significant differences in methodology, including study design and populations, a definite conclusion cannot be drawn, and further research is needed.

### Evaluating the differences between parents of children and/or youth who experience SITBs compared to parents in a comparison group

Four studies reported on the differences between parents of children and youth experiencing SITBs and parents in a comparison group [14, 49, 56, 62]. The study by James et al. [62] was an experimental cross-sectional study conducted among 60 mother-daughter dyads (ages ranging between 13 and 17) in which physiological markers (facial electromyography) were collected during standardized laboratory-based interaction tasks. In these tasks, dyads participated in a 2-min resting baseline task followed by positively-valanced (planning a dream vacation) and negatively-valanced (resolving a conflict) interactions. James et al. [62] compared the markers of mothers in dyads where youth experienced a history of NSSI to the markers of mothers in dyads where youth had no history of NSSI. Study results indicated that (1) mothers of youth with a history of NSSI exhibited elevated levels of negative facial expressions across tasks compared to control mothers, and (2) mothers of youth with a history of NSSI differed from control mothers in their patterns of self-reported anxiety across tasks, such that mothers of youth with a history of NSSI demonstrated higher levels of state anxiety following the negatively-valanced task and lower positive effect in response to the positively-valanced task relative to the control mothers. Finally, (3) mothers of youths with a history of NSSI were slower to return to their facial expression setpoint, demonstrating greater instability or fluctuations of facial affect.

Barksdale et al. [56] cross-sectional study was part of a more extensive national study involving 1854 children and youth (aged 5–22) and their parents that aimed to explore differences in their caregiver strain levels between parents of children and youth with no prior SAs, one prior SA, and multiple attempts. The results of Barksdale et al. [56] indicated that child lifetime SAs were associated with significant strain among caregivers. Further, they found that caregivers of suicidal children and youth experienced significantly more subjective internalizing strain (e.g., worry and guilt) and objective strain (e.g., interruption of personal time, lost work time, financial strain) but not subjective externalizing strain (e.g., anger, resentment, or embarrassment) compared to caregivers of non-suicidal children and youth. Moreover, pairwise contrasts of the groups indicated significant differences in subjective internalizing strain and objective strain between caregivers of children and youth with no vs. a single attempt, as well as between caregivers of children and youth with no vs. multiple attempts. It is important to note that the timing of the suicide attempt(s) relative to service entry and caregiver strain assessment was not clearly reported. Similarly, a mixed-methods study by Whitlock et al. [14] compared parental strain of parents of youth exhibiting NSSI to parents of youth with no known mental health history. Their study included 253 parents of youth aged 13–24 and found that scores on all measures of strain were higher among parents of NSSI youth compared to parents of youth with no known mental health history. Overall, having a self-injuring child explained a substantial proportion of variance between the two groups in all three categories of strain (objective, internalizing, and externalizing).

In contrast to those findings, Sheftall et al. [49] cross-sectional study compared 117 parents of children aged 6–9 with SI to parents of children who did not experience SI. The study results indicated that the parents groups did not differ significantly in their symptom levels (BSI).

In sum, our review indicates a significant difference in parental strain and distress between parents of children and youth who experience NSSI or SAs and those in a comparison group. Specifically, parents of children and youth with NSSI or SAs reported higher levels of strain and distress [14, 56, 62]. Only one study did not find a significant difference between parents of children experiencing SI and those whose children do not [49].

### Evaluating the effectiveness of support programs on parental stress and/or distress

The effectiveness of a support program (SPACE) specifically for parents of children and youth with DSH was evaluated in only one pre-post study (with no control group) [58]. SPACE is an eight-week group program that aims to guide parents on ways to provide emotional support to their child. The study included 46 parents of children and youth under the age of 16 who participated in the support program. The results indicated that the initial level of parental stress was high (GHQ-12: *M* = 6.33, SD = 4.1) and reduced significantly after program completion (GHQ-12: *M* = 0.88, SD = 2.47).

Eleven additional studies evaluated the effectiveness of therapeutic programs (including SAFETY, DBT, BRAVA, TORDIA, family therapy, and a specialized ER program for youth suicide attempts) designed to reduce SITBs among children and adolescents, and also reported data on parental change based on pre–post treatment design without a control group [7, 42, 44]– [45, 51, 63] or from between-group comparisons [43, 48, 52]– [53, 55]. Across both types of study designs, reductions in parental stress and/or distress symptoms were observed from pre- to post-program completion [7, 42–45, 51–53, 63], and in comparison, to a control group [48, 55]. The study by Wijana et al. [63] evaluated the effectiveness of a three-month course of family therapy for parents and was the only study that differentiated between mothers’ and fathers’ outcomes. This study included 49 parents of adolescents (aged 13–19) who had engaged in DSH or had expressed SI. Study results indicated that both mothers and fathers reported reduced levels of stress from pre- to post-therapy. Additionally, mothers experienced a significant decrease in anxiety symptoms over the course of the intervention. In contrast, fathers reported an increase in anxiety from pre- to post-therapy. However, this study did not include a control group, limiting the ability to draw causal conclusions about the observed changes. In sum, the studies reviewed indicate that both intervention programs aimed at reducing parental stress and those aimed at reducing youth SITBs led to a reduction in parental stress and distress from pre- to post-intervention. One study reported different outcomes for mothers and fathers [63], highlighting the need for greater inclusion of fathers in research.

## Discussion

Most of the research on children and youths’ SITBs has focused on identifying risk and protective factors associated with these behaviors or evaluating the effectiveness of interventions targeting youth. However, these studies have largely overlooked the potential association or impact of, children’s and adolescents’ SITBs on parental stress/distress. This scoping review aimed to address this gap, as a comprehensive overview and synthesis of the quantitative literature has been lacking. Twenty-five studies met our inclusion criteria. There was considerable variability in the measurement tools used, and only one study incorporated both self-report and physiological measures [62]. Most studies’ descriptive data indicated that parents experienced elevated levels of psychological stress and distress [e.g., 44, 57, 58, 63]. However, clinically elevated levels of depression, anxiety, or insomnia symptoms were reported only in some studies [e.g., 7, 54], while other studies reported symptoms that were sub-threshold [e.g., 61]. It is also important to note that some studies reported the percentage of parents who experienced clinically elevated levels of symptoms, while others reported the mean score. Additionally, the results suggest that using different questionnaires may yield varying outcomes. For example, the reviewed studies suggest that depression levels of parents, as measured by the CES-D, tended to be higher than those found in studies employing the BDI.

Our review indicates that two studies found a positive correlation between children’s and adolescents’ SITB and measures of parental stress and/or distress [50, 58], while two other studies found a partial association or reported evidence that could imply such an association [46, 59]. These results align with qualitative data suggesting that parents’ distress regarding their child intensifies when the child experiences SITBs [8]. Results also indicated that a child’s previous mental health history was associated with increased levels of parental distress- namely, depression and anxiety symptoms at follow-up [59], and lower parental well-being [57]. Only two studies did not identify any significant association between a child’s SI and NSSI and parental stress and/or distress, yet both reported a positive association between other clinical symptoms in the child/or youth and parental stress and/or distress [47, 61]. Variations in findings may stem from differences in measurement tools (e.g., different self-report questionnaires), illness severity (e.g., SI vs. DSH, NSSI, or SA; including a sample of children with SI is qualitatively different from a hospitalized sample of adolescents who have attempted suicide), variations in illness duration across studies, or a lack of information regarding the timing of the SITBs (past vs. current vs. unspecified) in some studies [56]. These features of the illness may affect parental stress and distress. For example, previous studies on parents of children with chronic physical conditions revealed that longer illness duration was associated with lower levels of parental stress and distress [e.g., 76

The results also revealed a notable increase in levels of parental stress and/or distress among those with children and/or youth engaging in NSSI or SAs when compared to parents in a control group [14, 56, 62]. Furthermore, it was found that mothers of youth with a history of NSSI exhibited higher levels of negative facial expressions compared to controls, along with increased instability or fluctuations in facial affect. However, contrary to these findings [14, 56, 62], one study found no difference between parents of children experiencing SI and those in a control group [49]. This latter study focused exclusively on parents of young children (aged 6–9) exhibiting SI. Collectively, the outcomes of these studies suggest that levels of parental stress and/or distress distinguish these parents from other groups of parents (whose children are not at risk for suicide). Notably, these results may be moderated by parent- and child-related factors. For example, age might moderate the effect of a child’s suicide risk on parental stress and/or distress, given that younger (younger than 10 years old) children’s deaths as a result of suicide are less frequent [1] and thus may be perceived as less threatening to parents relative to suicidality among youth, which results in more lethal outcomes. We advocate for future research to explore both parent- and child-related factors, comparing parents of children and/or youth experiencing SITBs with those of children and/or youth facing other mental illnesses that do not pose a threat to life. This approach will deepen our understanding of the repercussions of the threat to the child’s life on parental stress and/or distress.

Our results also indicate that all support programs or therapeutic interventions that assessed parental stress and/or distress (SAFETY; SPACE; DBT; BRAVA; TORDIA; FISP; family therapy, specialized ER care) led to a reduction in these symptoms from pre- to post-program completion [7, 42, 43, 43, 44, 44, 45, 51, 52, 52, 53, 63] and in comparison to standard care [48, 55]. This reduction in parental stress and/or distress levels following therapeutic intervention is encouraging and suggests that providing therapeutic attention and guidance to parents is beneficial. However, it is important to interpret these findings with caution, about half of the reviewed treatment studies assessed parental stress or distress from pre- to post-program completion without including a comparison group. Pre–post differences may be attributable to factors other than the intervention itself (e.g., placebo effects, regression to the mean), whereas findings from studies with a control or comparison group are less susceptible to such biases. Further, only one study assessed the differences between mothers’ and fathers’ outcomes after family therapy and found an increase in fathers’ reported anxiety from pre- to post-therapy [63]. The authors of this latter study suggested that as fathers were encouraged to take an active part in the therapeutic program, they shared the burden of care to a higher degree than usual. This increased involvement made them more aware of their child’s alarming symptoms, potentially leading to an increase in the fathers’ own distress or symptoms. In other words, fathers’ involvement in their child’s treatment, and not their gender, seemed to affect their distress levels. Focusing on children’s health, the National Academies of Sciences [77] recommended that research investigating the link between parental mental health and child’s mental health should encompass all primary caregivers, including fathers. Focusing on parents’ mental health, we propose that future studies should include male figures and further explore the distinctions between mothers and fathers, examining how current therapeutic guidance or treatments impact both parental figures based on their roles as primary or secondary caregivers.

Overall, the current scoping review revealed that the quantitative literature on parental stress and/or distress in response to their child’s SITBs is sporadic and lacking. Furthermore, with the exception of one study [58], many support programs or therapeutic interventions primarily involved parents as a means to prevent re-attempts or future SITB crises in their child, rather than focusing on the parents’ own well-being and burden of care. Consequently, information regarding what helped the parents is lacking. More broadly, our knowledge about parents’ stress and/or distress in the face of their child’s suicide risk remains very limited. We suggest here that examining the effects of children and/youth suicide risk on parents should be evaluated through the lens of stress-coping models [78]. Pearlin et al. [79], for example, suggested a stress-process model that describes how the interaction between caregiver variables (e.g., age, gender, culture), primary stressors (e.g., objective and subjective), secondary role strain (e.g., family conflicts, economic problems), secondary intrapsychic strain (e.g., global: self-esteem, mastery; situational: loss of self, role captivity), and mediating factors (e.g., coping, social support) affects caregiver outcomes (e.g., depression, anxiety, physical health). We suggest that future studies follow this model and explore how children and/or youth’s suicide risk affects parents’ stress and distress while taking into consideration caregiver variables (gender, age, culture, previous mental health history of the caregiver), primary stressors (e.g., illness-related factors such as suicide risk severity, duration, number of hospitalizations), secondary stressors (such as family conflicts, economic problems, as well as self-blame, anger, shame, helplessness, and self-efficacy in dealing with caregiving responsibility), and mediating factors.

Moreover, the current scoping review highlights that longitudinal data on parents of children and youth experiencing SITBs are limited and cannot support a causal association. Therefore, further longitudinal studies are required. Such longitudinal research will enable us to distinguish between different groups of parents and understand their adjustment or maladjustment processes, thereby evaluating the help they need more properly. To enhance the understanding of parental stress responses, future research should also incorporate real-time assessment methods, such as ecological momentary assessment (EMA) [e.g.,^80^], which would allow for capturing parents’ emotional and physiological responses in their natural environment. Additionally, more studies incorporating physiological measurements, such as heart rate variability (HRV) and cortisol levels, could provide objective indicators of parental stress and its potential long-term health consequences [e.g.,^81^].

### Limitations

Only 25 studies met our inclusion criteria, and many reported on parental stress and/or distress descriptively, as a secondary outcome, or as a covariate. As such, we were unable to perform a meta-analysis or compute effect sizes, a reflection of the existing literature, which is limited in size and mostly descriptive. While suicidal ideation and behaviors occur across various psychopathologies, we focused our search on depression, one of the strongest predictors of suicidal behavior [82]. However, including other disorders such as ADHD that has been recently acknowledged to be associated with increased risk for SITBs [83] in the search terms could have provided a broader perspective on parental stress and/or distress. Due to the lack of relevant literature, we broadened the definition of parental stress and/or distress to include studies using a wide range of self-report questionnaires that represent different psychological constructs. For example, some studies examined parental stress, anxiety, and depression, whereas others addressed parents’ caregiving strain, burden, or general well-being. Further, some studies evaluated children and/or youth’s suicide risk via parents’ self-report measures, whereas others used children and/or youth self-report measures. These differences may also have led to variability in results. Another limitation of this review is that some cross-sectional studies did not specify the timing of youth SITBs relative to parental stress and/ or distress assessment, limiting conclusions about their temporal relationship. Nevertheless, we contend that the merits of this study outweigh its limitations. In this scoping review, we focused on understanding how the child’s suicide risk is associated with parental emotional stress and/or distress, and it is the first review in which quantitative data were evaluated. The current findings support the idea that children’s and youth’s SITBs are associated with higher levels of parental stress and distress, suggesting new directions for understanding parental mental health.

### Implications

The study has several clinical implications. Specifically, the findings encourage the assessment of parental stress and/or distress levels associated with children and/or youth’s SITBs and the effect of intervention programs on parental outcomes. Relying on stress-coping models, we recommend that researchers examine the association between children and/or youth suicide risk and parents’ stress and/or distress while taking into consideration additional illness, caregiver, and mediating factors. Furthermore, we recommend that researchers conduct studies evaluating parental distress over time using diverse methodologies, with the aim of identifying trajectories of parental resilience and vulnerability, as well as identifying sensitive areas for therapeutic interventions. In addition, the current review suggests that future studies should consider examining the bidirectional association between children and/or youth’s SITBs and parents’ stress and distress, with precise consideration of symptom onset (timing), suicide risk severity, illness duration, and longitudinal follow-up of both child and parent. For example, it would be interesting to compare parents of youth who engaged in NSSI with parents of youth with suicidal intent or those who have attempted suicide. Further, we suggest therapists should be aware that, apart from their parental role as caregivers of their child, parents may also experience significant emotional distress that should be addressed and sometimes treated in therapy. Given the positive outcomes of various therapeutic interventions on parental distress discussed in this review, we strongly encourage therapists to involve parents in their children’s treatment.

## Data Availability

No datasets were generated or analysed during the current study.
